# Messenger RNA-based therapeutics for the treatment of apoptosis-associated diseases

**DOI:** 10.1038/srep15810

**Published:** 2015-10-28

**Authors:** Akitsugu Matsui, Satoshi Uchida, Takehiko Ishii, Keiji Itaka, Kazunori Kataoka

**Affiliations:** 1 Laboratory of Clinical Biotechnology, Center for Disease Biology and Integrative Medicine, Graduate School of Medicine, The University of Tokyo, Tokyo, 113-0033, Japan; 2 Department of Bioengineering, Graduate School of Engineering, The University of Tokyo, Tokyo, 113-8656, Japan; 3 Department of Materials Engineering, Graduate School of Engineering, The University of Tokyo, Tokyo, 113-8656, Japan

## Abstract

Gene therapy is a promising approach for treating diseases that are closely associated with excessive apoptosis, because the gene can effectively and sustainably introduce anti-apoptotic factors into cells. However, DNA delivery poses the risk of random genomic integration, leading to overexpression of the delivered gene and cancer development. Messenger RNA (mRNA) can evade integration events in target cells. We examined the use of mRNA-based therapeutics for introducing anti-apoptotic factors by using a mouse model of fulminant hepatitis. For introducing mRNA into the liver, a synthesised polymer-based carrier of polyplex nanomicelles was used for hydrodynamic intravenous injection. Using GFP as a reporter, we demonstrate that mRNA delivery induced efficient protein expression in almost 100% of liver cells, while plasmid DNA (pDNA) delivery provided a smaller percentage of GFP-positive cells. Analyses using Cy5-labelled mRNA and pDNA revealed that efficient expression by mRNA was attributed to a simple intracellular mechanism, without the need for nuclear entry. Consistent with this observation, Bcl-2 mRNA was more effective on reducing apoptosis in the liver of mice with fulminant hepatitis than Bcl-2 pDNA. Therefore, mRNA-based therapeutics combined with an effective delivery system such as polyplex nanomicelles is a promising treatment for intractable diseases associated with excessive apoptosis.

Gene therapy is a promising approach for the treatment of various intractable diseases, such as single-gene disorders, chronic disorders and cancers, because it can sustainably introduce therapeutic molecules in a target cell, and thus, directly regulate the cellular functions such as survival and (de)differentiation. Messenger RNA (mRNA) delivery has been shown to have several potential advantages over conventional gene delivery using DNA^1^,^2^,^3^. Gene therapy through mRNA delivery involves no risk of random genome integration, and can introduce protein into the cytoplasm without the need for nuclear entry, which leads to excellent protein production in any cell, including non-dividing cells^4^. Optimization of promoter is also not necessary, as is required with DNA delivery, and mRNA can rapidly express protein immediately after being introduced into the cells.

Although the small risk of random integration into genome is negligible in most cases of gene therapy, the risk becomes especially critical depending on the gene encoded by DNA, because the integration could lead to overexpression of the gene. For example, when DNA delivery was used for introducing signalling factors that induced critical effect on cell fates, e.g. factors related to cell survival, death, proliferation, and (de)differentiation, overexpression of such genes after integration could cause unexpected effect on the cells, which could lead to sever adverse effect on patients. Even when using vectors that have no integrase function, the risk of random integration cannot be completely prevented^5^,^6^. However, mRNA-based gene therapy can be a clinically effective means of introducing genetic components since it is theoretically ‘non-integrating’.

Anti-apoptotic factors are good candidates for gene therapy because various intractable diseases are closely associated with excessive apoptosis, where a substantial proportion of apoptotic cell death results in poor prognosis^7^,^8^. The introduction of mRNA encoding anti-apoptotic factors provides hope for achieving a clinically effective means of preventing cell death. If anti-apoptotic factors were introduced with DNA delivery, overexpression of the factors could lead to cancer development.

The purpose of this study is to test the feasibility of mRNA delivery for treating apoptosis-associated diseases. A mouse model of fulminant hepatitis was selected for evaluating the treatment efficacy of mRNA introduction of anti-apoptotic factors because the prognosis of hepatitis is closely related with apoptotic death of liver cells^9^. mRNA encoding Bcl-2, a representative anti-apoptotic factor, was administered *in vivo* into the liver, followed by histological analysis to evaluate the anti-apoptotic effect.

A major obstacle of *in vivo* delivery of mRNA to the liver is its susceptibility to nuclease-mediated degradation. One solution is to use a drug delivery system to transport the mRNA into the liver by preventing its degradation. Polyplex nanomicelles consisting of a poly(ethylene glycol) (PEG)-coated surface and an mRNA-containing core is a promising system because the nanomicelles provide excellent *in vivo* stability of nucleic acids including plasmid DNA (pDNA), small interfering RNA (siRNA), and mRNA, under physiological conditions^10^,^11^,^12^,^13^. Furthermore, the stealth property provided by the polyplex nanomicelle surface, composed of dense PEG palisades, effectively evades host immune defenses^10^,^14^.

To maximise mRNA uptake into liver cells, hydrodynamic tail vein injection was applied. This technique facilitates efficient cellular uptake of nucleic acids, typically of pDNA, into mouse liver by increased hydrodynamic pressure^15^,^16^. Although the hydrodynamic injection induced transient damage in liver tissue^17^, this technique allows comprehensive uptake into cells in the target organ, and would be most suitable for analyzing the intracellular processes of mRNA as compared with pDNA. Once mRNA is safely internalised into the liver cells, it is expected to induce rapid and efficient effects on the cells compared with pDNA administration, because mRNA delivery does not require the process of nuclear entry.

In this study, we first evaluated gene expression after hydrodynamic injection of mRNA or pDNA using gene reporter assays, with detailed investigation into the expression profiles in the liver tissue. We then tested an anti-apoptotic therapy using mRNA encoding Bcl-2 in model mice with fulminant hepatitis.

## Results

### Distribution of GFP expression in the liver after administration of GFP-expressing mRNA or pDNA

Protein expression profiles after hydrodynamic injection of mRNA or pDNA into liver tissue were evaluated using GFP as a reporter. Polyplex nanomicelles loaded with mRNA or pDNA encoding GFP were prepared by mixing the PEG-polycation block copolymer with mRNA or pDNA solution, as previously described^10^,^13^,^18^. For the cationic segment of the block copolymer, we used poly[N′-[N-(2-aminoethyl)-2-aminoethyl] aspartamide] (PAsp(DET)), which has low cumulative toxicity because of its self-catalytic degradation property^19^,^20^. We performed chemical modification of mRNA, which involved substitution of cytidine (C) and/or uridine (U) with 5-methyl-C and/or pseudo-U (ψU), because it has been reported to enhance mRNA mediated protein expression efficiency after *in vitro* and *in vivo* mRNA delivery^21^,^22^,^23^. To optimise the condition of mRNA modification for hydrodynamic injection into the liver, several formulations of the modified mRNA were tested. The formulation in which C was 100% substituted with 5-methyl-C exhibited the highest level of GFP expression in the liver (Supplementary Fig. S1). Thus, this formulation was used for subsequent experiments.

**Figure 1:**
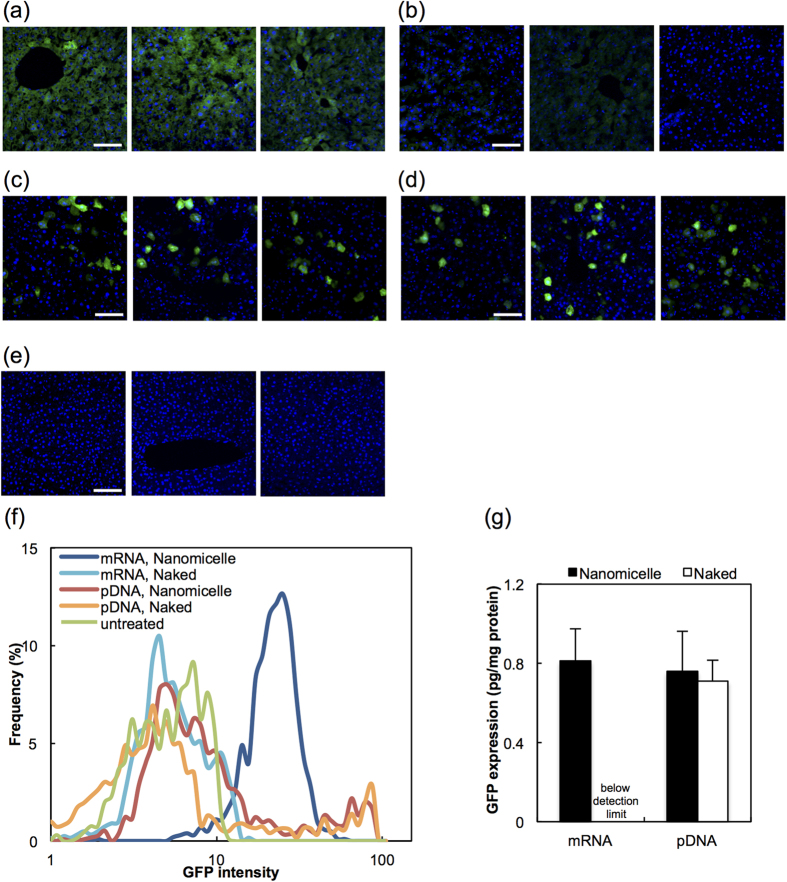
GFP expression in liver tissue after hydrodynamic injection of mRNA or pDNA. GFP expression in the liver was evaluated 24 h after hydrodynamic injection of GFP-expressing mRNA or plasmid DNA (pDNA). (**a–f**) Confocal laser scanning microscopy images of liver tissues were obtained after immunohistochemical staining for GFP (green) and staining of cell nuclei with DAPI (blue). (**a**) mRNA-loaded polyplex nanomicelle, (**b**) naked mRNA, (**c**) pDNA-loaded nanomicelle, (**d**) naked pDNA, (**e**) untreated. Scale bars: 100 μm. The intensity of GFP fluorescence was quantified using image analysis software and presented in a histogram in (**f**). Twelve images from three mice in each group were used for the analyses. (**g**) GFP expression was measured with an ELISA using liver tissue homogenates. The expression after delivery of naked mRNA was below the detection limit. The data are presented as the mean ± standard error of the mean (s.e.m.) (N = 4).

Since the naked form of pDNA has been reported to induce high transgene expression after hydrodynamic injection^15^,^16^, mRNA and pDNA were injected in their naked forms, as well as in the nanomicelle-loaded form. Uniformly distributed GFP signals were observed in liver tissue, in almost all liver cells, under confocal microscopy 24 h after injecting nanomicelles loaded with mRNA encoding GFP (Fig. 1a,f). Injection of the mRNA in the naked form (i.e. not loaded into the polyplex nanomicelle) provided almost no GFP expression (Fig. 1b). In contrast, injecting pDNA showed a remarkably different profile of GFP expression, in which the number of GFP-positive cells was significantly lower, but the intensity of GFP signals in individual cells was greater than injection with mRNA-loaded nanomicelles (Fig. 1c,d). GFP expression measured from liver tissue homogenates by ELISA showed comparable values for mRNA and pDNA, suggesting that the total amount of protein production in liver tissue was equivalent (Fig. 1g).

### Comparison of mRNA and pDNA

As shown in the previous section, mRNA needs to be loaded into polyplex nanomicelles to provide sufficient protein expression in the liver. Accordingly, the introduction of erythropoietin using nanomicelles loaded with mRNA induced a significant hematopoietic effect (Supplementary Fig. S2), whereas the naked mRNA showed almost no effect. It is interesting that, in the case of pDNA delivery, the naked form induced GFP expression comparable to that of nanomicelle-encapsulated pDNA (Fig. 1g). To investigate this difference between mRNA and pDNA, the protein expression processes of the both delivery mechanisms following *in vivo* hydrodynamic injection were analysed in detail.

The degradation profiles of mRNA and pDNA were evaluated by measuring the residual copies of mRNA or pDNA encoding GFP in liver extracts using quantitative real-time PCR (qRT-PCR). The copies were quantified at 10 min after injection as it is reported that uptake of nucleic acids to liver cells is completed within 10 min post hydrodynamic injection^24^. The results are represented as percentages of the injected mRNA or pDNA dose (Supplementary Fig. S3). The obtained values are highly correlated with the expression competency of mRNA and pDNA since this method allows for the detection of mRNA and pDNA in which the sequences between the primers remain intact. The amount of mRNA that was detected in the liver after injecting mRNA-loaded nanomicelles was significantly higher than that observed using naked mRNA. In contrast, in the case of pDNA, similarly high amounts of remaining pDNA were detected in the liver tissue after injection with both naked pDNA and pDNA-loaded nanomicelles. These results suggest that mRNA is more susceptible than pDNA to enzymatic degradation, and the nanomicelles effectively prevented degradation of the mRNA *in vivo*. The residual amount of mRNA in the liver after injecting mRNA-loaded nanomicelle was similar to that of pDNA after injecting naked pDNA or pDNA-loaded nanomicelle (Supplementary Fig. S3).

**Figure 2:**
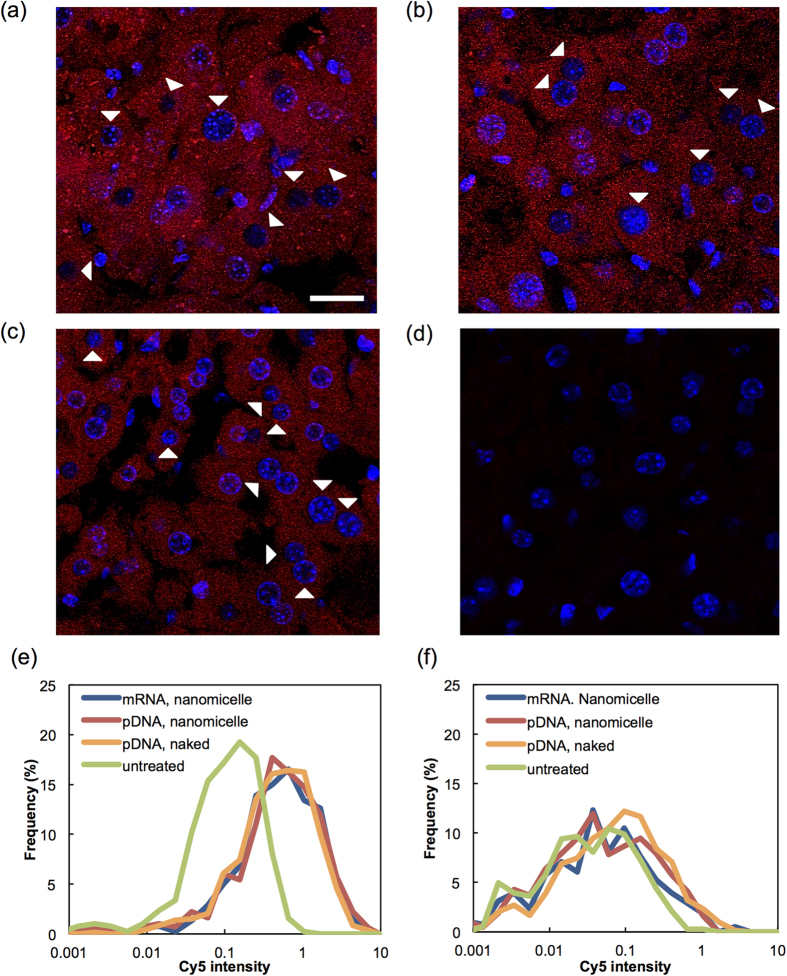
Distribution of injected mRNA and pDNA in liver tissue. Confocal laser scanning microscopic images of liver tissues were obtained 30 min after hydrodynamic injection of Cy5-labeled mRNA and pDNA (red). (**a**) mRNA-loaded polyplex nanomicelle, (**b**) pDNA-loaded nanomicelle, (**c**) naked pDNA, (**d**) untreated. Cell nuclei were stained with DAPI (blue). Arrowheads indicate nuclei with inefficient uptake of Cy5-labeled mRNA or pDNA. Scale bars: 20 μm. (**e,f**) The intensity of Cy5 in the cytoplasm (**e**) and the nucleus (**f**) was quantified using image analysis software and presented in a histogram. Twelve images from three mice in each group were used for the analyses.

To investigate the reason for the different profiles in the GFP expression between mRNA and pDNA (Fig. 1a–f), we analysed cellular uptake and intracellular processing using Cy5-labelled mRNA and pDNA. The fluorescent images of the liver cells were obtained using a confocal laser-scanning microscope 30 min after injecting mRNA-loaded nanomicelles, pDNA-loaded nanomicelles, or naked pDNA. Labelled mRNA or pDNA appeared to be distributed in the cytoplasm in almost 100% of liver cells (Fig. 2a–d), showing no significant difference among the three groups. However, the Cy5 signal in the nucleus was much lower than that in the cytoplasm for both mRNA and pDNA, and was almost undetectable in a substantial percentage of cells. These findings were confirmed by quantifying Cy5 signals in the cytoplasm and nucleus using image analysis software (Fig. 2e,f). Considering the simple intracellular mechanism of mRNA to provide protein expression in the cytoplasm without the need for nuclear entry, it is reasonable to assume that the mRNA injected by the nanomicelles produce protein in the cytoplasm of almost 100% of liver cells, as previously demonstrated in Fig. 1a. In contrast, since nuclear transport was required for pDNA delivery, the ratio of transgene expression-positive cells by pDNA injection was significantly decreased despite the similar distribution profile as mRNA (Fig. 1c,d).

**Figure 3:**
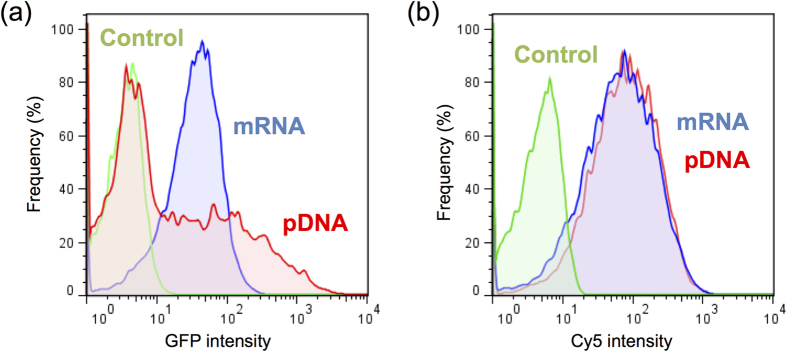
GFP expression and cellular uptake of mRNA and pDNA in cultured cells evaluated by flow cytometry (FCM). GFP-expressing mRNA or pDNA was delivered into HuH-7 cells using a *Trans*IT Transfection system for FCM analyses. (**a**) GFP expression in each cell presented in a histogram. To obtain the maximum expression in each group, mRNA-treated cells and pDNA-treated cells were analysed at 24 h and 48 h after delivery, respectively (see Supplementary Fig. S4). (**b**) The cellular uptake efficiency of mRNA or pDNA presented in a histogram. FCM analyses were performed at 24 h after transfection of Cy5-labeled mRNA or pDNA. Blue: mRNA, red: pDNA, green: untransfected.

These results strongly suggest that nuclear entry would be a critical obstacle of pDNA injection, highlighting the advantage of mRNA to provide protein expression. This feature is also confirmed by an analysis using culture cells. Flow cytometry (FCM) of HuH-7 cells was performed after transfection of mRNA or pDNA encoding GFP using the *Trans*IT Transfection system at the time point in which GFP expression from each delivery mechanism reached its maximum value; 24 h for mRNA and 48 h for pDNA (Supplementary Fig. S4). Transfection of mRNA provided GFP expression in almost 100% of cells (Fig. 3a). In contrast, the transfection efficiency for pDNA transfection was approximately 50%, although the peak GFP intensity in the histogram was remarkably higher than that after mRNA transfection. Quantification of the efficiency of cellular uptake using Cy5-labelled mRNA or pDNA showed that nearly 100% of cells were Cy5-positive for both mRNA and pDNA (Fig. 3b). These results strongly suggest that not all of the internalised pDNA would result in expression of the transgene due to the low efficacy of nuclear transport. These findings using culture cells completely correlated with the observations in liver cells (Fig. 1), and further confirm the advantage of mRNA for providing high efficiency of transgene expression to nearly 100% of cells because of its simple intracellular mechanism.

### Therapeutic application of mRNA delivery

**Figure 4:**
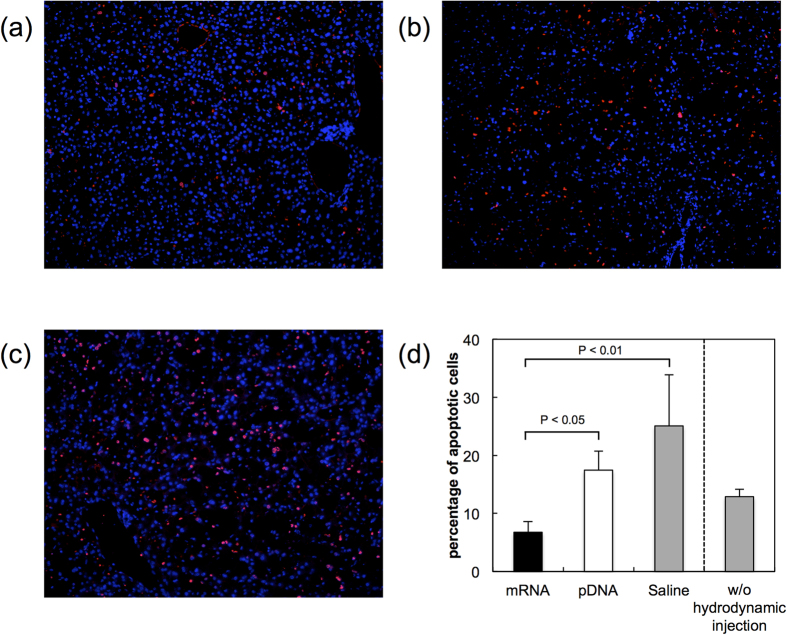
Introduction of Bcl-2 in a mouse model of fulminant hepatitis. A mouse model of fulminant hepatitis was prepared by intraperitoneal injection of Fas ligand. Fifteen minutes after the injection, Bcl-2 was introduced into the liver through hydrodynamic tail vein injection using mRNA-loaded nanomicelles or naked pDNA. Four hours after the injection, liver sections were prepared for TUNEL staining to visualise apoptotic cells (red). Cell nuclei were stained with DAPI (blue). (**a–c**) Representative images after the injection of (**a**) mRNA, (**b**) pDNA, or (**c**) saline. (**d**) Percentage of apoptotic cells in total liver cells in each image. The percentage of apoptotic cells in the liver of Fas-ligand-injected mice without hydrodynamic injection is also shown. Five or more images were analysed for each mouse. Six mice were used for mRNA-, pDNA- and saline-treated groups, and 4 mice were used for the group without hydrodynamic injection. The data are presented as the mean ± standard error of the mean (s.e.m.). Statistical analysis was performed using one-way analysis of variance followed by Tukey’s multiple comparison tests.

Finally, the nanomicelle-based mRNA delivery was applied for treating fulminant hepatitis, which was induced by intraperitoneal injection of Fas-ligand. This disease is strongly associated with massive apoptosis of hepatocytes. mRNA or pDNA encoding a representative anti-apoptotic protein, Bcl-2, was administered by hydrodynamic injection using polyplex nanomicelles. The TUNEL staining assay for identifying apoptotic cells in liver tissue demonstrated that the number of apoptotic cells was reduced 4 h after injection of mRNA encoding Bcl-2 compared to saline-treated controls (Fig. 4a,c). Injection of pDNA encoding Bcl-2 also inhibited apoptosis, although to a much lesser extent than mRNA encoding Bcl-2 (Fig. 4b). Quantification of the apoptotic cells confirmed that the percentage of apoptotic cells in liver tissue was decreased by mRNA-Bcl-2 injection compared to the control without hydrodynamic injection (Fig. 4d). Although it appears from the saline control that the hydrodynamic injection technique was invasive to further increase the number of apoptotic cells than that of the group without injection, mRNA mediated introduction of Bcl-2 significantly reduced apoptosis compared with other groups receiving hydrodynamic injection of Bcl-2 pDNA or saline, indicating that mRNA was of advantage in exerting the anti-apoptotic effect presumably due to its high efficiency in transgene expression in the liver.

## Discussion

In this study, we investigated the feasibility of mRNA delivery for introducing cell signalling proteins. Despite several advantages of mRNA delivery, reports of *in vivo* mRNA delivery are still quite few^3^. A major obstacle to *in vivo* mRNA delivery is its susceptibility to nuclease-mediated degradation. Therefore, in this study, we encapsulated mRNA into polyplex nanomicelles to prevent degradation^10^,^13^. The mRNA-loaded nanomicelles were effectively internalised in the liver cells following hydrodynamic injection into the liver, and provided a high yield of transgene expression (Fig. 1, Supplementary Fig. S2). Interestingly, whereas almost no GFP expression was detected by injecting naked mRNA, pDNA delivery resulted in efficient protein expression equivalently for pDNA-loaded nanomicelles and the naked pDNA. Although naked pDNA has commonly been used for hydrodynamic injection in previous studies^15^,^16^,^25^, we previously reported that the encapsulation of pDNA into nanomicelles could reduce the tissue damage associated with hydrodynamic injection in the skeletal muscle^26^,^27^,^28^. In this study, there were no significant differences between naked pDNA and pDNA loaded into nanomicelles, suggesting that pDNA was sufficiently stable to reach the liver after hydrodynamic injection, and that the nanomicelle formulation did not interfere with the intracellular process of transgene expression.

In striking contrast, mRNA needs to be loaded into the nanomicelles to achieve transgene expression in the liver. This is apparently attributed to the fact that mRNA is much more susceptible to nuclease-mediated degradation than pDNA. Thus, for *in vivo* mRNA delivery, the delivery system for stably carrying the mRNA is critical for achieving efficient protein expression, and the polyplex nanomicelle system proved to be a promising solution.

The homogeneous expression pattern in the target tissue was a critical aspect of mRNA delivery in this study. Microscopic observation of GFP expression in the liver demonstrated that mRNA delivery induced uniform expression in almost 100% of liver cells, whereas pDNA delivery provided a small percentage of GFP-positive cells using either the naked form or pDNA-loaded polyplex nanomicelles. The difference in the expression profile is probably due to differences in the intracellular processes for protein expression between mRNA and pDNA. We analysed the cellular uptake using Cy5-labelled pDNA or mRNA and determined that although the uptake into the cytoplasm was achieved in almost 100% of liver cells for both pDNA and mRNA, their nuclear localisation was limited to a relatively smaller number of cells (Fig. 2). Since pDNA needs to be transported into the nucleus to express protein, the limited nucleus entry is the probable cause of the low number of GFP-positive cells by pDNA transfection (Figs 1 and 3). In contrast, mRNA is capable of producing the protein in the cytoplasm, thus providing a higher ratio of transgene-positive cells.

Moreover, as indicated by histograms (Fig. 1f for *in vivo* and Fig. 3a for *in vitro*), the expressed GFP intensity was distributed within a narrow range by mRNA transfection. This remarkably homogeneous profile of transgene expression by the mRNA strongly suggests that the intracellular process of protein translation by the mRNA would not be the rate-limiting step, but once the mRNA is internalised into the cytoplasm, protein translation would be equally initiated in each cell.

It is interesting that the total amount of protein production in the liver was equivalent between pDNA and mRNA, as assessed by ELISA (Fig. 1g). Considering the relatively low ratio of transgene-positive cells by pDNA transfection, it can reasonably be assumed that the expression level in individual cells would fluctuate considerably, and the peak level of transgene expression in individual cells was considerably higher than that observed with mRNA transfection. When the purpose of gene therapy is to provide secretory proteins or peptides, where the introduced factors should be efficiently secreted even from very few cells, pDNA introduction would be a suitable delivery mechanism for gene therapy.

In contrast, when applying cell signalling proteins and peptides, the homogeneity of transgene expression by mRNA would be of great advantage in achieving therapeutic effect. Since the signal transduction would be regulated in a very sensitive and specific manner in individual cells, it is essential to introduce the signaling factors uniformly into the greatest possible number of cells. The results of mRNA introduction into the liver clearly indicate the potential of mRNA-based gene therapy for regulating cellular survival and functions by introducing signalling proteins and peptides.

Among the signalling factors, an anti-apoptotic factor, Bcl-2, was chosen here for demonstrating the therapeutic efficacy of mRNA introduction. When applying mRNA encoding Bcl-2 in a mouse model of fulminant hepatitis, the number of apoptotic liver cells showed a significant decrease compared to the saline control (Fig. 4), while pDNA encoding Bcl-2 induced only a minimal therapeutic effect to prevent apoptosis. Since the total protein production in the liver tissue was comparable between mRNA and pDNA, the superior therapeutic effect by mRNA to prevent apoptosis is probably primarily due to the uniformly distributed homogeneous transgene expression profile achieved by the hydrodynamic injection into the liver. Since the hydrodynamic injection technique is invasive to further increase the number of apoptotic cells in the liver of fulminant hepatitis model, a more sophisticated technique for introducing mRNA into the liver cells is required for applying the therapeutic strategy for human patients. Nevertheless, the results obtained here suggest the potential of mRNA for the purpose of regulating the cell survival and functions by introducing cell signalling factors.

It is generally difficult to introduce the factors by proteins and peptides directly into cells, because it is difficult to penetrate the lipid bilayer of the cell membrane to reach intracellular spaces while maintaining their tertiary structures. Other problems associated with direct introduction of proteins into cells include instability in blood and other extracellular spaces, short duration of action, and the high cost. In this study, it is revealed that mRNA-based gene therapy is a reasonable alternative for protein delivery. The polyplex nanomicelles play a key role in stably and effectively delivering the mRNA into the target cells. The mRNA can provide transgene expression in a uniformly distributed homogeneous manner, which would be of great advantage especially for introducing the signalling factors. Since the mRNA can express any protein and peptide by modifying the sequences, this system can be adapted for various therapeutic purposes involving controlling cells.

In conclusion, the hydrodynamic injection of mRNA encoding Bcl-2 with polyplex nanomicelles into the liver of mice with fulminant hepatitis effectively reduced apoptosis of the liver cells. In contrast, pDNA encoding the same factor induced only a minimal therapeutic effect, although the total protein production in the liver tissue was comparable to that of the mRNA. This difference in therapeutic outcome between mRNA and pDNA can be attributed to a difference in the efficiency of protein expression since mRNA delivery resulted in protein expression in almost all cells in the liver, as determined by GFP analysis. In contrast, a relatively smaller percentage of cells were GFP positive after pDNA delivery, although the peak GFP intensity was higher than that observed with mRNA delivery. These results demonstrate the feasibility of mRNA-based therapeutics combined with an effective delivery system using polyplex nanomicelle for the treatment of intractable diseases associated with excessive apoptosis.

## Methods

### Preparation of mRNA and pDNA

mRNA was prepared through *in vitro* transcription (IVT) using a MEGAscript T7 Transcription Kit (Ambion, Austin, TX, USA). pDNA templates for IVT were constructed from the pAcGFP vector (Clontech, Mountain View, CA, USA) for GFP, the pORF-mEPOv18 vector (Invitrogen, Carlsbad, CA, USA) for erythropoietin, and the pCMV-XL4-Bcl2 vector (OriGene, Rockville, MD, USA) for Bcl-2. The coding region of each vector was inserted into the pSP73 vector (Promega, Madison, WI, USA) for expression under the T7 promoter. To attach a poly(-A) chain to the mRNA 3′ terminal, a 120-bp poly A/T sequence was cloned into the pSP73 vector down-stream of the protein coding sequence. For chemical modification of mRNA, 5-methyl-cytidine (5-methyl-C) and/or pseudo-uridine (ψU) (TriLink, San Diego, CA, USA) was added to the IVT reaction solution to substitute for cytidine (C) and uridine (U), respectively. mRNA prepared through IVT was purified using an RNeasy Mini Kit (Qiagen, Hilden, Germany). pDNA for *in vitro* and *in vivo* administration was prepared by inserting the coding regions of the pAcGFP vector and the pCMV-XL4-Bcl2 vector into the pCAG-GS vector (Riken, Tokyo, Japan). Cy5 labelling of mRNA or pDNA was performed with a Label IT Nucleic Acid Labelling Kit, Cy™5 (Mirus, Madison, WI, USA). The concentration of pDNA and mRNA was determined spectroscopically at 260 nm.

### Preparation of polyplex nanomicelles loaded with mRNA and pDNA

The PEG-poly[N’-[N-(2-aminoethyl)-2-aminoethyl]aspartamide] [PEG-PAsp(DET)] block copolymer was synthesised according to a previous report^19^. The molecular weight of PEG was 12,000. The polymerisation degree of the PAsp(DET) segment in ^1^H-NMR analysis was 57. Nanomicelles were prepared as described previously^10^. Briefly, the PEG-PAsp(DET) polymer and nucleic acids (mRNA or pDNA), separately dissolved in 10 mM HEPES buffer (pH 7.3), were mixed by rapid pipetting. The N/P ratio [(Total amines in polymer)/(mRNA phosphates)] was 3. The final concentration of nucleic acid was adjusted to 33.3 μg/ml for all samples. The size of the nanomicelles loaded with mRNA and pDNA was 50 nm and 90 nm, respectively, when measured with dynamic light scattering as in previous studies^10^,^18^.

### Hydrodynamic injection

Using Balb/c mice (7 weeks old, female; Charles River Laboratories, Yokohama, Japan), hydrodynamic injection was performed as reported previously^15^,^25^. In brief, solutions containing 5 μg of mRNA or pDNA, prepared as described above, were diluted with saline to obtain 1.8 ml of solution, which was injected into the tail vein of a mouse weighing around 23 g in 5 sec. All animal studies were approved by the Animal Care and Use Committee of the University of Tokyo, Tokyo, Japan, and were performed in accordance with the guidelines for care and use of laboratory animals of the University of Tokyo.

### Enzyme-linked immunosorbent assay (ELISA) of GFP expression efficiency in liver

Liver tissue was homogenised with a Multi-Beads Shocker (Yasui-kiki, Osaka, Japan) 24 h after hydrodynamic injection. ELISA was performed using a GFP ELISA kit (Abcam, Cambridge, UK) as described in the manufacturer’s protocol. The amount of GFP was standardised to the amount of total protein in the homogenates; measured using the Micro BCA Protein Assay Kit (Thermo Fisher Scientific, Waltham, MA, USA).

### Evaluation of the hematopoietic effect

To evaluate haematopoiesis, 100 μl of blood was collected after submandibular bleeding, as described in a previous report^29^. The collection was performed at 28 d and 56 d after the introduction of erythropoietin. Haematocrit and haemoglobin values were measured with pocH-100i (Sysmex, Hyogo, Japan).

### Quantification of injected mRNA and pDNA in the liver

Copies of injected nucleic acids (mRNA or pDNA) were measured with quantitative real-time PCR (qRT-PCR). Liver tissue was homogenised with a Multi Beads Shocker. Total RNA and DNA were purified using an RNeasy Mini Kit and a DNeasy Blood & Tissue Kit (Qiagen), respectively. qRT-PCR was performed using an ABI Prism 7500 Detector (Applied Biosystems, Foster City, CA, USA) and a primer pair for the GFP sequence (forward, CAACTACAACGCCCACAATG and reverse, GTTGTGGCGGATCTTGAAGT), with standardisation to the amount of β-actin (Mm00607939, TaqMan Gene Expression Assays; Applied Biosystems). The graph indicates the percentage of nucleic acids in the liver per total injected dose.

### Observation of GFP expression and Cy5-labeled mRNA and pDNA in liver sections

Mouse liver was perfused with phosphate buffered saline (PBS; Wako Pure Chemical Industries, Osaka, Japan) and 4% paraformaldehyde (PFA) in PBS (Wako). The tissue was then incubated with 4% PFA in PBS overnight, with 10% sucrose in PBS for 4 h, with 15% sucrose for 4 h, and 20% sucrose overnight at room temperature. The samples were frozen in optimal cutting temperature compound (Sakura Finetek, Tokyo, Japan). Frozen sections were prepared at a thickness of 10 μm. The sections were treated with rabbit anti-GFP IgG (Invitrogen) at a 1:300 dilution for 2 h and then with Alexa 488 goat anti-rabbit IgG (Invitrogen) for 1 h at room temperature. Samples were mounted in ProLong Gold Antifade Reagent with DAPI (Invitrogen). Images were acquired using an LSM 510 confocal microscope (Carl Zeiss, Oberkochen, Germany) with a 20× objective at excitation wavelengths of 488 nm (Ar laser) for GFP, 633 nm (He-Ne laser) for Cy5, and 710 nm (MaiTai laser for 2-photon imaging) for DAPI. From these images, GFP expression and the cellular uptake of Cy5-labeled mRNA and pDNA into the cytoplasm and nucleus were evaluated using image analysis software (IN Cell Analyzer Workstation 3.7.1; GE Healthcare, Buckinghamshire, UK). The area of the nucleus was defined as the area stained with DAPI, and that of cytoplasm was defined as the area within 10 μm from the edge of the nucleus.

### *In vitro* assays using flow cytometry (FCM)

HuH-7 cells were seeded onto 6-well plates at a density of 100,000 cells/well and cultured in Dulbecco’s Modified Eagle Medium (Sigma-Aldrich, St. Louis, MO, USA) containing 10% foetal bovine serum (FBS; Dainippon Sumitomo Pharma, Osaka, Japan) and penicillin-streptomycin (Sigma-Aldrich). After 24 h of culture, the medium was replaced with Opti-MEM (Invitrogen) without serum, and GFP-expressing mRNA or pDNA was added to each well (2.5 μg/well) using *Trans*IT mRNA Transfection Reagent (Mirus) for mRNA, and *Trans*IT-LT1 transfection Reagent (Mirus) for pDNA. At 4 h after transfection, the medium was replaced with DMEM containing 10% FBS. GFP expression and the cellular uptake of Cy5-labelled mRNA or pDNA were quantified with a BD™ LSR II Flow Cytometer (BD Biosciences, San Jose, CA, USA).

### Fulminant hepatitis

The mouse model of fulminant hepatitis was prepared by intraperitoneal injection of Jo-2, a Fas-ligand (BD Biosciences) at a dose of 5 μg/mouse^30^,^31^. Liver tissue sections were prepared as described above and subjected to terminal deoxynucleotidyl transferase dUTP nick end labelling (TUNEL) staining using the *In Situ* Cell Death Detection Kit, TMR red (Roche, Basel, Switzerland) to identify apoptotic cells. Fluorescence images were acquired with an IN Cell Analyzer 1000 (GE Healthcare) and analysed using IN Cell Analyzer Workstation 3.7.1 software.

## Additional Information

**How to cite this article**: Matsui, A. *et al.* Messenger RNA-based therapeutics for the treatment of apoptosis-associated diseases. *Sci. Rep.* **5**, 15810; doi: 10.1038/srep15810 (2015).

## Supplementary Material

Supplementary Information
