# Supplementary material for: Messenger RNA-based therapeutics for the treatment of apoptosis-associated diseases
Source: Sci Rep. 2015 Oct 28;5:15810. doi: 10.1038/srep15810 (PMC4623474; doi:10.1038/srep15810)
Supplement: Supplementary Information [file srep15810-s1.pdf]

Supplementary Figures

Messenger RNA-based therapeutics for the treatment of apoptosis-associated diseases

Akitsugu Matsui, Satoshi Uchida, Takehiko Ishii, Keiji Itaka, Kazunori Kataoka

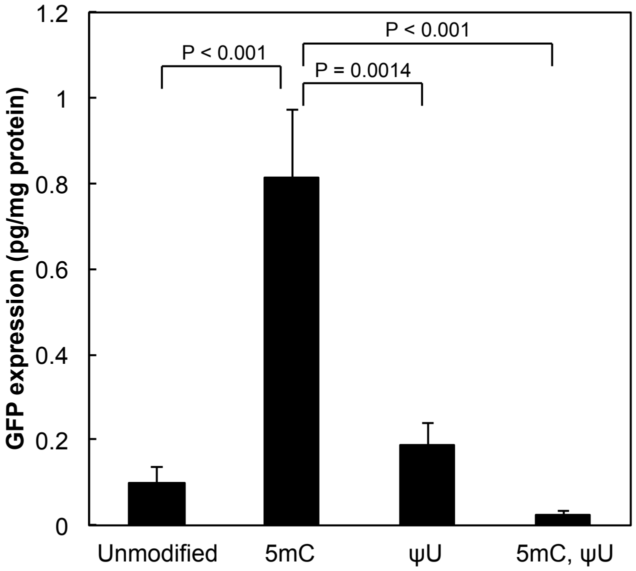

Supplementary Figure S1. Optimisation of mRNA modifications based on the GFP expression efficiency in the liver. mRNA modifications were optimised using unmodified mRNA and modified mRNA with 100% substitution of cytidine (C) and/or uridine (U) with 5-methyl-C (5mC) and/or pseudo-U (ψU), respectively. Modified mRNAs were hydrodynamically injected using polyplex nanomicelles, and GFP expression was measured with an ELISA using liver tissue homogenates 24 h post injection. The data are presented as the mean ± standard error of the mean (s.e.m.) (N = 4). Statistical analysis was performed using one-way analysis of variance followed by Tukey's multiple comparison tests.

(a)

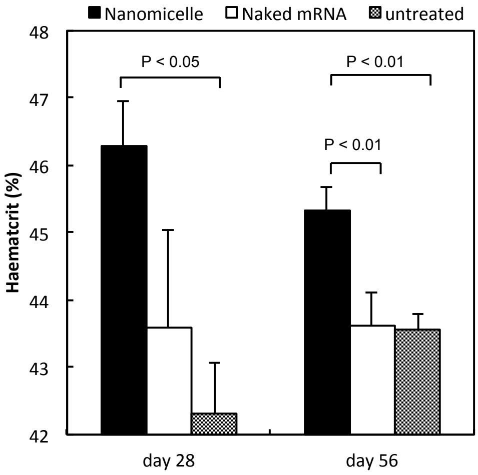

(b)

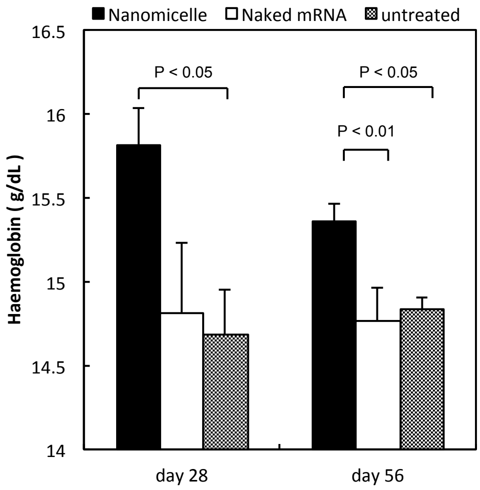

Supplementary Fig. S2. Haematopoiesis after hydrodynamic injection of erythropoietin-expressing mRNA. Erythropoietin-expressing mRNA was hydrodynamically injected into mouse liver as naked mRNA or in polyplex nanomicelles. (a) Haematocrit and (b) haemoglobin. N = 8, for nanomicelle-treated and untreated mice, and N = 6, for naked mRNA-treated mice. The data are presented as the mean ± standard error of the mean (s.e.m.). Statistical analysis was performed using one-way analysis of variance followed by Tukey’s multiple comparison tests.

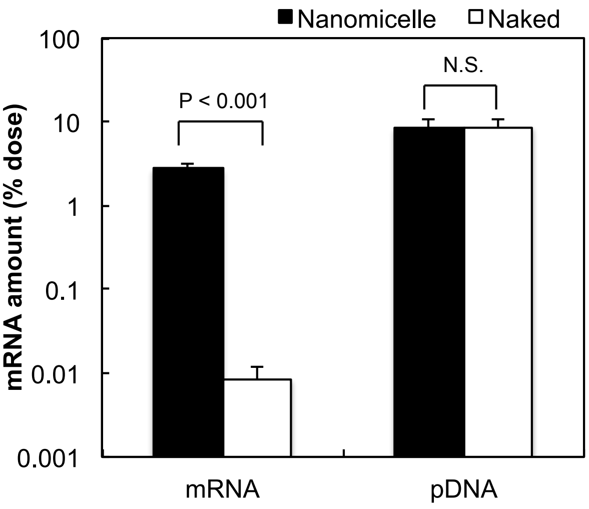

Supplementary Fig. S3. Degradation of mRNA or pDNA after hydrodynamic injection measured with quantitative real-time PCR (qRT-PCR). GFP-expressing mRNA or pDNA was hydrodynamically injected into the mouse liver as naked mRNA or in polyplex nanomicelles. At 10 min after the injection, the total mRNA or pDNA were extracted from the liver, and the copy number of GFP-expressing mRNA or pDNA in the liver was measured with qRT-PCR. In the graphs, a value of 100% represents a case in which all injected nucleic acids distributed to the liver and remained intact. The data are presented as the mean ± standard error of the mean (s.e.m.) (N = 5). Statistical analyses were performed using an unpaired two-tailed Student's t-test.

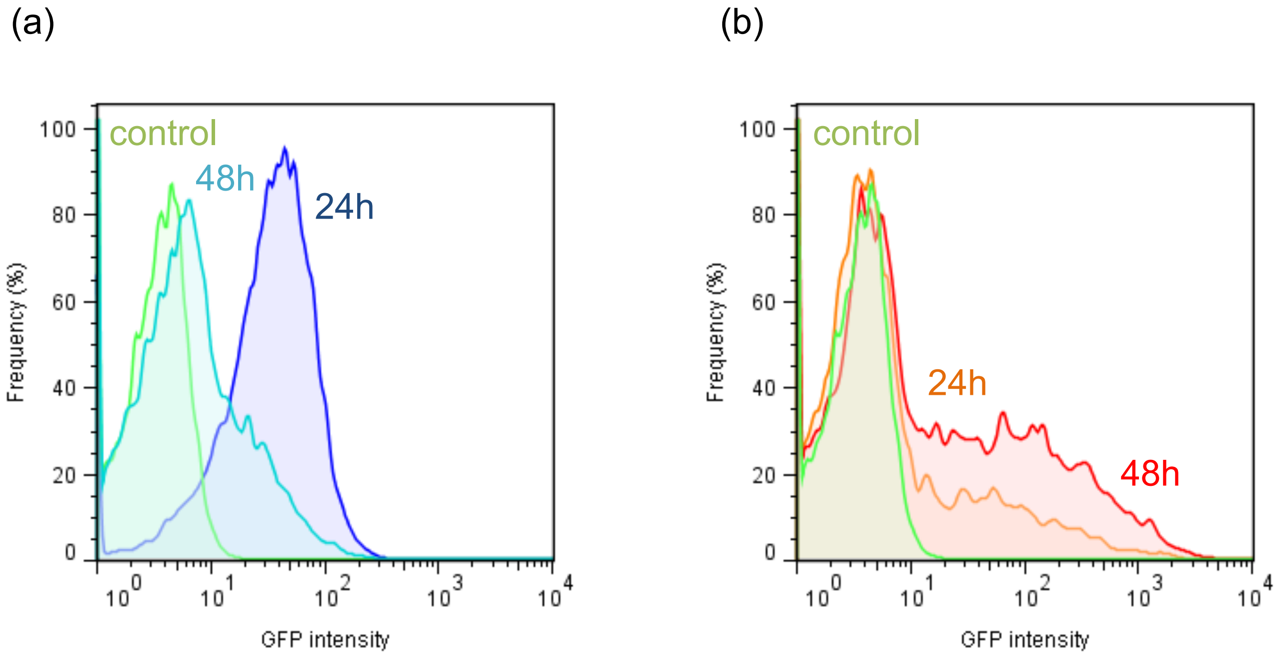

Supplementary Fig. S4. Time-dependent profile of GFP expression. GFP-expressing mRNA (a) or pDNA (b) was delivered into HuH-7 cells using a *Trans*IT transfection system. Flow cytometry analyses were performed 24 h and 48 h after transfection. GFP expression in each cell is presented in a histogram.
